# The independent and combined effect of price increase and price labelling on sugar-sweetened beverage vending machine sales in an Australian university: a factorial randomised controlled trial

**DOI:** 10.1186/s12966-026-01890-4

**Published:** 2026-03-18

**Authors:** Miranda R. Blake, Jane Dancey, Adrian J. Cameron, Anna Peeters, Julie Brimblecombe, Liliana Orellana

**Affiliations:** 1 Deakin University, Institute for Health Transformation, Locked Bag 20000, Geelong, VIC 3220 Australia; 2 Deakin University, Global Centre for Preventive Health and Nutrition, School of Health and Social Development, Institute for Health Transformation, Faculty of Health, Locked Bag 20000, Geelong, VIC 3220 Australia; 3 Department of Nutrition, Dietetics and Food, Monash University, 264 Ferntree Gully Rd, Notting Hill, VIC 3168 Australia; 4 Deakin University, Biostatistics Unit, Faculty of Health, Locked Bag 20000, Geelong, VIC 3220 Australia

**Keywords:** Sugar-sweetened beverages, Vending, Universities, Health promotion, Australia

## Abstract

**Background:**

No studies have examined how price increases on unhealthy drinks in university vending machines impact customer purchasing or vendor financial implications. Vending machines often display prices only upon product selection, hindering consumers’ price evaluation.

**Objective:**

To assess the independent and combined effects of (i) a 20% price increase on unhealthy drinks, and (ii) prominent labels displaying the price below each product, on the healthiness of customer purchases and revenue in university vending machines.

**Design:**

Two-year 2 × 2 factorial cluster randomized controlled trial (March 2020 to February 2022). Machines were clustered by proximity. Itemised weekly sales data were collected for 33 weeks pre-trial and 103 weeks during the trial. Six-monthly audits assessed treatment maintenance.

**Participants/setting:**

Sixty vending machines across four campuses of a university in Victoria, Australia, with 59 machines analyzed.

**Intervention:**

Machine groups were randomly allocated to four conditions: (i) 20% price increase on unhealthy drinks (‘Price Increase Only’); (ii) prominent labels displaying the price below each product (‘Price Label Only’); (iii) ‘Price Increase + Price Label’; and (iv) no price increase or labels (‘Control’).

**Main outcome measures:**

The primary outcome was the change from pre-trial mean of volume percentage of unhealthy drinks sold per machine per week. Secondary outcomes included volume percentage of healthier drinks sold per week and revenue.

**Statistical analyses:**

Sales data were analysed using linear mixed models with machine cluster and machine as random effects; and fixed effects for price, label, price × label, pre-trial mean outcome, teaching period, COVID-19 lockdowns, and season.

**Results:**

Mean volume percentage of unhealthy drink sales was higher for the ‘Price Label Only’ compared to ‘Control’ condition (6.34 percentage points, 95%CI: 1.85, 10.82), percentage of unhealthy drink units sold, and energy and total sugar content of drinks sold, and lower for volume and unit percentage of unhealthy drink sales. No other significant differences were observed for the primary or secondary outcomes.

**Conclusions:**

This study in university vending machines found no impact of a 20% unhealthy drink price increase, and an increase in volume sales of unhealthy drinks in response to prominent price labelling only.

**Trial registration:**

Australian New Zealand Clinical Trials Registry Number: ACTRN12620000206921 (prospectively registered 20 February 2020).

**Supplementary Information:**

The online version contains supplementary material available at 10.1186/s12966-026-01890-4.

## Background

Vending machines offer consumers 24/7 convenience for a “delivery on demand” era [1]. Hospitals, universities and travel hubs are key settings for testing vending market innovations [1]. Universities present an opportunity to promote improved nutrition in young people via health-promoting food and drink vending machine interventions. Recent reviews within and outside the university setting have found that vending machine interventions that increase healthy food availability and reduce the price of healthy alternatives are likely to be most effective at increasing the healthiness of consumer purchases [2–4]. We are aware of four published studies testing vending machine pricing interventions in universities [5–8]. French et al. [5], found an 80% increase in sales of healthy snacks with a 50% price discount in a US university, and no overall change in total number of snacks sold. They did not report revenue or profit outcomes. Hua et al. [6] in an 8-arm factorial randomised controlled trial found discounts of 25–50% on healthy foods and drinks in a US university were not associated with changes to purchases on their own, but price discounts alongside increased availability of healthy products and decreased availability of unhealthy products, or healthy signage advertising healthy products, increased revenue and increased healthiness of purchases. Lambert et al. [8] in a pre-post study in US university halls of residence found that 25% price discounts on healthy snacks combined with an increased availability of healthy snacks was associated with increased percentage of these items purchased, with total snack sales decreasing over the intervention period. Price discount alone or snack availability changes alone were not tested. While many healthy vending machines interventions have been identified in recent reviews [2–4], only one study examining the effect of price increases on unhealthy products has been reported to date [7]. A non-randomised trial in a US university tested a multicomponent intervention with a 25% price increase on chocolate bars, stickers identifying healthy options, and improved relative availability and placement of healthy snack options [7]. They found eight times more healthier products purchased in intervention compared to control machines, no change to revenue, and increased profit compared to a control condition [7]. Studies to date have mostly been conducted in the US and have varied in quality and size, with no published studies having examined price increases on unhealthy drinks in vending machines over more than one year.

Beyond the vending market, raising prices on unhealthy options is an increasingly popular strategy for influencing consumer choices, as demonstrated by the number of jurisdictions adopting sugar-sweetened beverage (SSB) taxes globally [9, 10]. Such taxes have been shown to drive industry reformulation and consumer behaviour change, and are frequently directed by government finance departments based on their potential to generate revenue, as well as public health objectives [11]. In retailer-led pricing interventions, reductions in overall unit sales due to increased prices on unhealthy products may be offset by the resulting greater profit margins on unhealthy products [12]. This may help overcome the fear of profit loss which often deters suppliers and retailers from pricing-based healthy food interventions [12]. Business outcomes of interventions are likely to be of particular interest to vending operators, as global and Australian vending machine market revenue is not expected to equal pre-COVID-19 levels until at least 2027 [1, 13].

Evidence suggests that the convenience aspect of vending may lower price sensitivity with consumers willing to pay more at vending machines than other retail formats [14]. Displaying all product prices at once in a vending machine (increasing “price salience”) theoretically facilitates product comparisons necessary to promote the healthier options where they are cheaper than less healthy options [15]. However, some newer vending machines, especially those with interactive screen displays, do not display prices directly below the product. Instead, the price is only displayed on the selection screen for a selected item. This industry trend could therefore reduce price salience and impact the effectiveness of pricing interventions.

This study aimed to assess the combined and independent effects of (i) a 20% price increase on unhealthy drinks and (ii) prominent pricing labels, on the healthiness of customer purchases and revenue in university vending machines.

We hypothesised that:

- A 20% price increase on unhealthy drinks would reduce purchases of unhealthy drinks, with no effect on overall vending revenue.
- Prominent price labelling alone would have no effect on vending purchasing or revenue.
- Prominent price labelling, combined with a 20% price increase on unhealthy drinks, would reduce purchases of unhealthy drinks over and above the additive effects of price increase and price labelling alone, and have no effect on vending revenue.

## Methods

### Setting

Monash University is a large Melbourne-based university with several campuses spread across Greater Melbourne and internationally. In 2020 (year of trial commencement), there were 16,708 staff and 75,394 onshore students across Victorian campuses with wide ranging disciplines [16]. Monash has a complex food environment, with chain and independent café-style and fast-food outlets, grocery stores and vending machines. Across the campuses, there were 60 vending machines with Western-style snacks and drinks, in addition to 44 vending machines with Asian-style snacks and drinks. All machines had a combination of both snacks and drinks. In the Western-style machines, prior to the intervention, prices were digitally displayed at the payment terminal only when the customer entered the item number, with no price labels displayed next to the physical product. In the Asian-style machines, prices were displayed directly below the physical product.

In October 2018, Monash reached full implementation of the voluntary Victorian state Government *Healthy Choices* guidelines [17] for vending machines. The guidelines use food categories, portion size and nutrient criteria to classify food and drinks into ‘green’ (‘best choice’, e.g., sparkling water), ‘amber’ (‘choose carefully’, e.g., small flavoured milk), and ‘red’ (‘limit’, e.g., full sugar soft drinks). These guidelines specify that healthiest (‘green’) food and drinks should comprise at least 50% of available items, and least healthy (‘red’) food and drinks should comprise no more than 20% of available items. Moderately healthy ‘amber’ items should comprise the remainder of options. The terms ‘red’, ‘amber’ and ‘green’ are used hereafter.

### Trial design

A parallel 2 × 2 factorial cluster randomised controlled trial (RCT) (1:1:1:1 allocation ratio) was conducted.

### Participants

All combined snack and drinks vending machines on Monash University Victorian campuses were eligible for inclusion. Two campuses also provide student accommodation, some of which contain vending machines. The supplier of the Asian-style vending machines declined to participate at the time of the study. The focus of the current intervention was the 60 Western-style vending machines.

### Interventions

Prior to the intervention, no price labels were displayed below products in any vending machine. A 2 × 2 factorial design was used to test the independent and combined effect of two interventions: a 20% price increase on ‘red’ drinks (‘Price Increase’), and prominent price labels (price per unit) added below all products at the point-of-selection (‘Price Label’). The four conditions defined by the combination of these two interventions were (i) ‘Price Increase Only’; (ii) ‘Price Label Only’; (iii) ‘Price Increase + Price Label’; and (iv) neither price labels nor pricing changes (‘Control’).

**Fig. 1:**
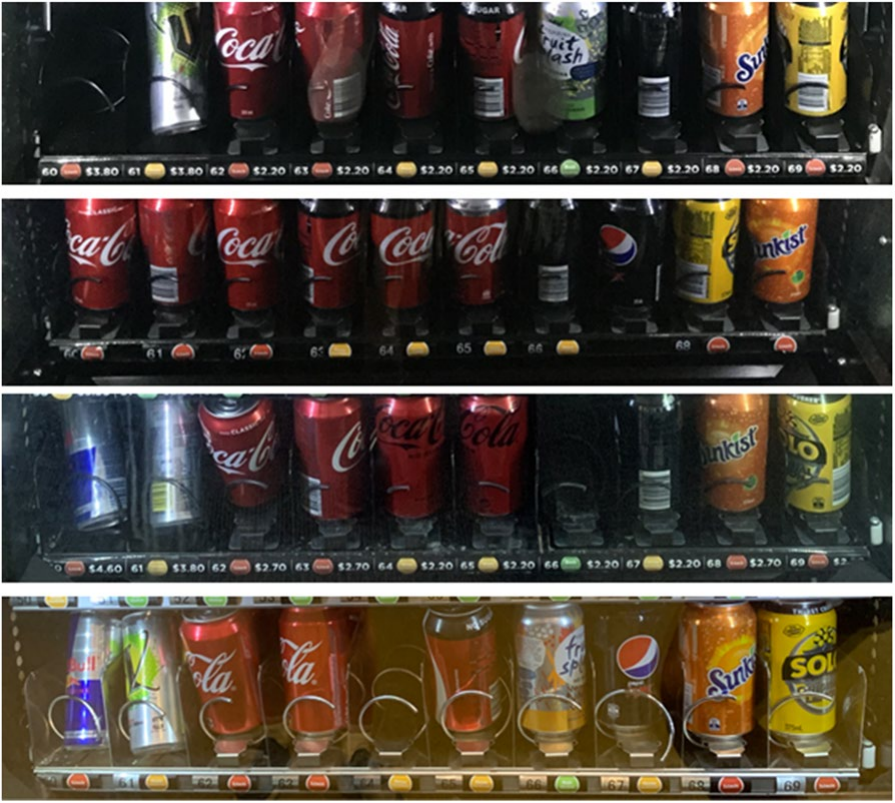
Example photos of vending machines conditions. From top to bottom: **a** ‘Price Label Only’; **b** ‘Price Increase Only’ (price not visible); **c** ‘Price Increase + Price Label’; and **d** ‘Control’ conditions (no price increase or label)

Price labels measured 12 mm x 300 mm to fit machine specifications and were placed adjacent to the existing product number and circular sticker of the product *Healthy Choices* guidelines traffic light classification with words ‘best choice’ (‘green’), ‘choose carefully’ (‘amber’) or ‘limit’ (‘red’). The total label size was 20 mm x 700mm. A 20% ‘red’ drink price increase was selected as it aligns with the World Health Organization’s minimum recommended SSB taxation to meaningfully reduce consumption [18]. Increased ‘red’ drink prices for intervention machines were rounded up to the nearest AU$0.10, to ensure that prices were at least 20% higher than at baseline (e.g., $3.70 + 20% rounded up to $4.50 (21.6% increase)). Vending machine changes were implemented between 11–13 March 2020. The intervention period lasted 103 weeks from 9 March 2020 (beginning of Semester 1, 2020) to 27 February 2022 (prior to start Semester 1, 2022). Figure 1 provides example photographs of the conditions including label positions.

### Outcome measures

All outcomes were defined per machine per week. Volume and units sale outcomes were defined as the difference between the volume/unit percentage sold in each week and the mean of the volume percentage sold across the pre-trial period. In what follows we will refer to these outcomes as to “percentage volume/units change from baseline”. A similar definition was used for energy, total sugar sold and revenue from drinks.

The primary outcome was percentage volume change from baseline of ‘red’ drinks. Secondary outcomes included change from baseline in: percentage volume of ‘amber’ and ‘green’ drinks; percentage units of ‘red’, ‘amber’ and ‘green’ drinks sold; energy (kJ/100mL) and total sugar (g/100mL) from drinks; machine revenue from snacks and drinks (AUD); and total number of ‘red’, ‘amber’ and ‘green’ snack units.

### Data collection

#### Sales data

Weekly itemised drink and snack sales data for each machine were obtained from the vending supplier from 22 July 2019 (33 weeks prior to the intervention and the earliest date available due to change in sales data management system) until 27 February 2022 (end of intervention). Data included week start and end date, product name, product sale price and number of units of each product sold. We did not have access to cost price of items, and could therefore not calculate profit data.

#### Nutritional content of products sold

For each snack and drink product available, the vending supplier also provided drink product volume (ml) or snack weight (g) per package; Victorian state government voluntary ‘*Healthy choices’* guidelines [17] classification (‘green’, ‘amber’, ‘red’); and nutritional content (information as per nutrition information panels, i.e. energy, protein, fat, saturated fat, carbohydrate, total sugar, sodium per 100 g). Undergraduate nutrition students independently collected nutrition information from manufacturer or supermarket websites (where available) and confirmed product *‘Healthy choices*’ traffic light classifications via the FoodChecker online *‘Healthy choices’* food classification database [19]. Supplier and student classifications and nutrition information agreed for all items.

#### Auditing

Photographs of all included vending machines were taken at 6-monthly intervals during the RCT: at 1 month (March 2020), 6 months (August 2020), 12 months (February 2021), 18 months (November–December 2021) and 24 months (February 2022). This photographic audit and an audit survey of machines were used to determine implementation and maintenance of the intervention, as well as to collect contextual information to be used as covariates in the analysis. Compliance with the intervention was assessed at each audit, including: whether prominent price labels were present on all items; price per unit for regular ‘Coke’ and ‘Coke No Sugar’ (as examples of widely available drinks which should have different prices if the price increase was correctly applied). Contextual information collected included whether machines were (i) located in a student residential area, (ii) co-located with Asian-style vending machines not subject to the trial; or (iii) had a touchpad machine user interface (affecting price display). Photographs were taken to allow verification of survey data. Audits were completed by research staff via the Qualtrics survey platform on tablets or mobile phones. See Appendix I for full survey.

### Sample size

Sample size was based on pragmatic considerations. All available Western-style vending machines in the Victorian Monash University campuses were included in the study.

### Randomisation and blinding

Machines were clustered by precinct, so that vending machines in close proximity (e.g. within a library) were allocated to the same condition to avoid people moving to a nearby machine with cheaper prices. Next, machine clusters were assembled into four groups, with characteristics balanced across groups, prioritised in the described order: (i) representation of machines from each campus; (ii) size of clusters; (iii) mean total units sold in machines in January 2020; and (iv) machine ranking of sales volume of units sold in January 2020 from 1 (highest sales) to 60 (lowest sales). Each group of machines was randomly allocated (using random numbers generated in Microsoft Excel) to one of the four intervention conditions by a statistician blind to machine groups. This strategy was used instead of randomisation of machine clusters to balance the above-described characteristics across intervention conditions.

The analyst was blind to group allocation. It was not possible to blind customers to the interventions, but customers were not explicitly notified of any intervention or that the vending machines were part of a study.

### Statistical analysis

Separate analyses were conducted for each outcome of interest. The weekly time series for each outcome was divided in two periods: pre-intervention or ‘baseline’, 22 July 2019 to 8 March 2020 (33 weeks); and ‘intervention’ 9 March 2020 to 27 February 2022 (103 weeks). Due to the large variability of sales across machines, weekly outcomes were calculated as the difference between the weekly value during the intervention period and the average baseline level of that outcome across the 33 weeks.

For each outcome, a linear mixed model with cluster and machine as random effects and autocorrelation lag 1 was fitted. The model included as fixed effects the two intervention indicators (‘price’ and ‘label’) and the interaction price × label. Models were fitted additionally adjusting for: mean outcome (over the pre-intervention period) to further control for sale variability across vending machines, machine location in a student residential area (indicator variable), season (Summer, Autumn, Winter, Spring), number of days university semester was in session per week (1 to 7) [20–22], and number of days per week state government COVID-19 lockdown ‘stay at home’ orders were in place (1 to 7) [23]. For each outcome we report three pre-planned contrasts estimated from the adjusted model: each intervention condition against control condition, alongside 95% confidence intervals and the interaction price × label p-value. For the main outcome (percentage of ‘red’ drinks sold by volume) we also report unadjusted estimates.

#### Sensitivity analyses

**Fig. 2:**
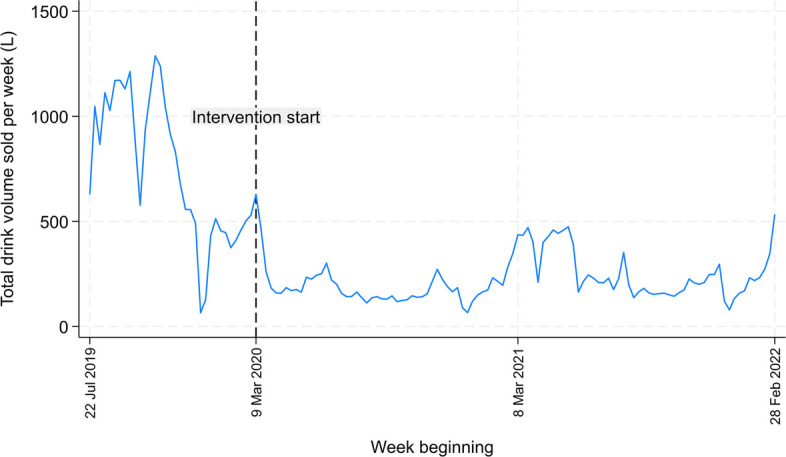
Total volume sales of drinks over time for all vending machines (22 July 2019 to 6 March 2022)

Sensitivity analyses were conducted for the primary outcome (percentage volume change from baseline of ‘red’ drink volume sold per week) using adjusted linear mixed models as described above. The first sensitivity analysis was a per protocol analysis reflecting actual label and pricing conditions received for two machines that incorrectly did not receive any intervention during the trial period. The second sensitivity analysis excluded sales data from 9 March 2020 to 7 March 2021; this period was marked by very low sales when most staff and students were absent from campus due to COVID-19 stay-at-home orders. Figure 2 demonstrates the total volume sales of drinks over time for all machines.

### Changes in analysis plan from registered protocol

Originally, a one-year intervention was planned (from March 2020 to February 2021). The start of the intervention period coincided with beginning of the COVID-19 stay-at-home orders (March 2020). From March 2020-October 2021, Melbourne, Victoria had a total of 262 days with stay-at-home orders in place [23]. The research team and vending company therefore made the decision at the time to expand the intervention period to two years (March 2020 to February 2022) to ensure that sufficient customers would be on-campus to avoid very low sales affecting analysis results.

We had also planned to conduct an interrupted time series analysis [24, 25]. However, less than one year of pre-intervention data was available, so we were unable to model seasonal trends required for this approach. Also, as the beginning of the RCT coincided with the beginning of the COVID-19 lockdown, the core assumption required for that method that prior trends would be sustained during the intervention period was clearly violated.

Finally, we had planned to adjust for campus enrolment data (number of students on each campus per semester). Given that enrolment was not reflective of campus attendance during the COVID-19 period, we instead included number of days semester was in session each week, and number of days COVID-19 ‘stay-at-home’ orders were in place each week.

### Ethical approval

A low-risk project approval was obtained from Deakin University Human Ethics Advisory Group, Faculty of Health (reference HEAG-H 111_2019). Monash University Human Research Ethics Committee provided recognition of prior ethical approval (reference 23,084). No consent or assent was necessary or obtained for the vending machine intervention from customers.

### Trial registration

The trial was registered with Australian New Zealand Clinical Trials Registry (ANZCTR) prior to commencement on 20 February 2020, ACTRN12620000206921.

## Results

**Fig. 3:**
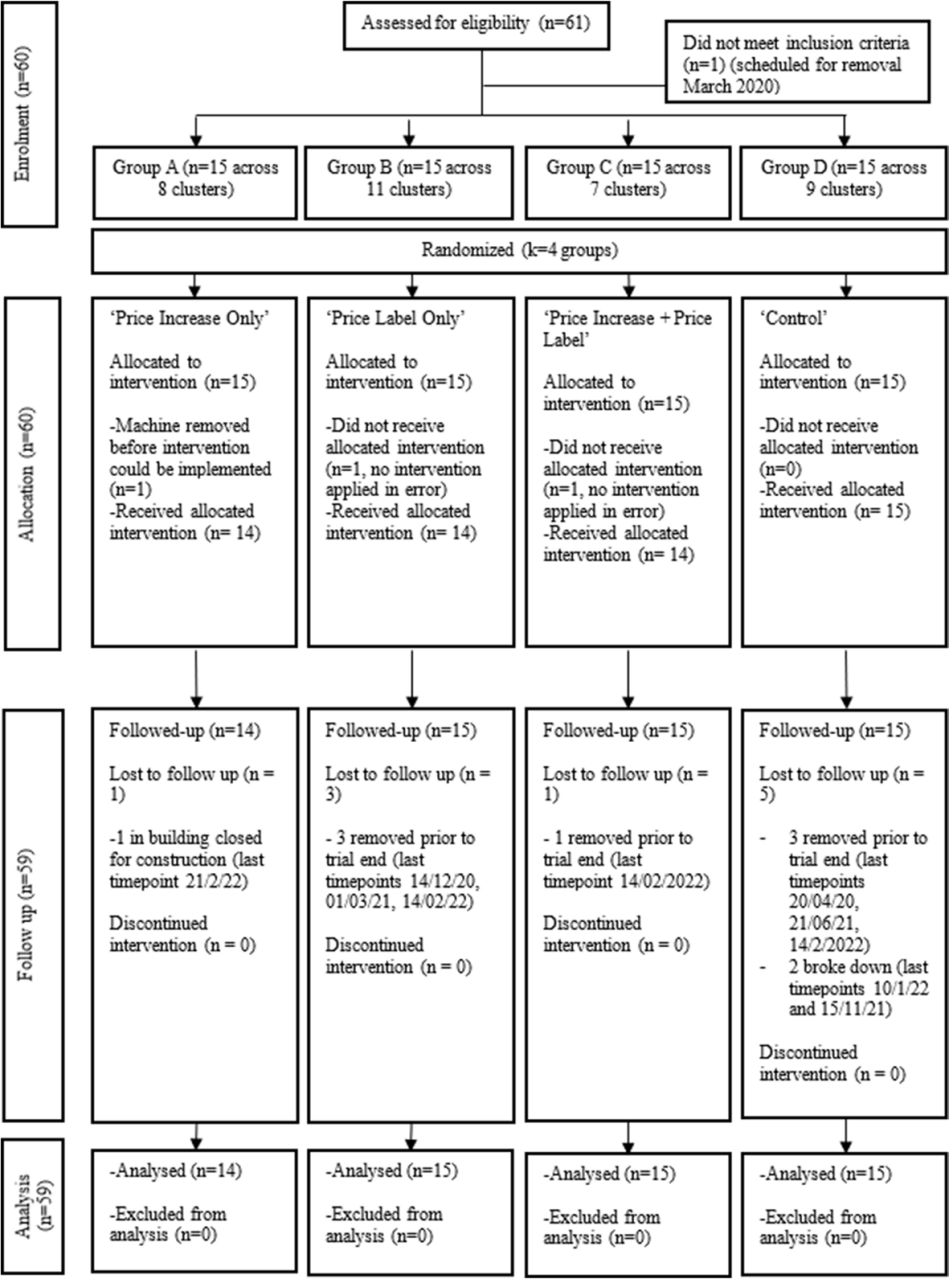
CONSORT flow diagram for university vending pricing trial, 2020 to 2022

The CONSORT flow diagram displays the number of vending machines included at each stage of the trial (Fig. 3). Of the 60 machines randomised, data from 59 machines were analysed in the intention-to-treat approach, excluding data from one machine removed before the intervention period began. Audits identified that two intervention machines did not receive the allocated condition (instead received no intervention). These machines were at isolated off-campus locations. Subsequent audits confirmed that all other 57 allocated operational machines were consistent in their pricing and labelling conditions for all 6-monthly audit timepoints until February 2022, except for ten machines that were removed or located in a building closed for construction during some of the trial period, 5 of which were removed during the final two months of the trial.

All characteristics were balanced across conditions at baseline except for touchpad user interface (7 machines) (Table 1). No significant differences were found in baseline weeks of data, percentage of drink volume sold at baseline classified as ‘red’. Total drink volume sales over time within each treatment condition is shown in Supplementary Figure S1. The mean number of weeks of data during the intervention period (SD) was similar across conditions: ‘Price Increase Only’ (96.2 (20.3)), ‘Price Label Only’ (103.9 (0.27)), ‘Price Increase + Price Label’ (103.7 (0.59)) and ‘Control’ (93.5 (25.8)) (*p* = 0.2870). Our analysis did not adjust for co-location of Asian-style vending machines and touchpad interface as these infrequently occurred within our sample (Table 1).

**Table 1:** Overview of vending machine baseline characteristics by treatment condition of university pricing trial (*n* = 60)

	Overall (*n *= 60 machines)	Price Label Only (*n* = 15 machines)	Price Increase Only (*n* = 15 machines)	Price Increase + Price Label (*n* = 15 machines)	Control (*n* = 15 machines)	*p*-value
***n* (%)**						
Number of clusters (% of total)^a^	37	8 (22%)	11 (30%)	7 (19%)	9 (24%)	0.213^b^
Co-location with Asian-style vending machines	3 (5%)	0 (0%)	0 (0%)	1 (7%)	2 (13%)	0.605^c^
Located within student residential area	14 (23%)	4 (27%)	3 (20%)	3 (20%)	4 (27%)	1.000^c^
Touchpad machine user interface	7 (12%)	0 (0%)	2 (13%)	0 (0%)	5 (33%)	0.012^c^
Campus locations						
Main metropolitan campus	42 (70%)	10 (67%)	11 (73%)	11 (73%)	10 (67%)	0.593^b^
Other locations	18 (30%)	5 (33%)	4 (27%)	4 (27%)	5 (33%)	
** median (range) **						
Number of machines per cluster^a^	2 (1–5)	2 (1–3)	2 (1–3)	3 (1–5)	2 (1–3)	0.2760^d^
**mean (SD) **						
Total snack, drink and other units sold^e^	657.4 (431.9)	660.1 (485.4)	746.2 (465.8)	697.5 (482.3)	525.6 (250.8)	0.7567^d^
Sales volume ranking^f^	32.5 (18.3)	32.7 (21.9)	29.3 (18.5)	31.7 (19.7)	36.3 (13.6)	0.7573^d^
Weeks of machine observations at baseline	325 (2.2)	32.3 (2.6)	32.3 (2.6)	32.3 (2.6)	33.0 (0.0)	0.7928^d^
** mean (95% CI)**						
Percentage of ‘red’ drinks sold by volume per week at baseline^g^	27.9 (24.5, 31.2)	32.0 (24.9, 39.1)	26.6 (20.5, 32.6)	28.1 (21.0, 35.2)	25.4 (18.3, 32.5)	0.5759^b^
Millilitres of all drinks sold per week at baseline^h^	11,740 (9339, 14,143)	11,653 (6507, 16,800)	12,190 (7466, 16,913)	11,955 (7000, 16,911)	10,848 (5573, 16,122)	0.9849^b^

^a^Clusters were defined as machines in close geographical proximity such as in the same building

^b^Chi-squared test of comparison across groups

^c^Fisher’s exact test of comparison across groups

^d^Kruskal Wallis test of comparison across groups

^e^Sales per machine in month of January 2020

^f^Machines were ranked by number of units sold in January 2020 from 1 (highest sales) to 60 (lowest sales)

^g^Unadjusted linear mixed model estimates

^h^To convert to fluid ounces, divide by 29.57

### Primary outcome

The unadjusted mean change from baseline in weekly percentage of ‘red’ drinks sold was not different from zero in any of the four conditions with no significant differences relative to control condition (Table 2, Unadjusted). However, the adjusted estimate showed a significant decrease in percentage volume of ‘red’ drinks sold during the intervention period for the ‘Price Increase Only’ condition (−5.92 [95% CI −9.03, −2.80]), the ‘Price Increase + Price Label’ (−3.87 [−7.03, −0.71]) and the ‘Control’ (−3.78 [−6.93, −0.622]) conditions. There was a significant 6.34 percentage point increase (95%CI 1.85, 10.82) in the adjusted mean percentage volume change of ‘red’ drinks sold in the ‘Price Label Only’ condition relative to the Control condition, with no other significant differences versus ‘Control’ (Table 2, Adjusted).

**Table 2:** Change from baseline in weekly percentage of ‘red’ drinks sold for each condition (*n* = 59 machines)

Analysis	Estimated mean change from baseline in weekly sales (95%CI)				Difference relative to control condition in weekly sales (95%CI)			Interaction Price × Label
	Price Increase Only	Price Label Only	Price Increase + Price Label	Control	Price Increase Only	Price Label Only	Price Increase + Price Label	p-value
Main analyses								
Unadjusted^a^	−4.05 (−8.94, 0.84)	1.56 (−3.74, 6.85)	−3.42 (−8.73, 1.89)	−2.08 (−7.67, 3.50)	1.24 (−8.12, 10.61)	6.66 (−3.38, 16.7)	2.74 (−7.28, 12.77)	0.576
Adjusted^b^	−5.92 (−9.03, −2.80)^c^	2.56 (−0.51, 5.63)	−3.87 (−7.03, −0.71)^d^	−3.78 (−6.93, −0.622)^d^	−2.14 (−6.57, 2.29)	6.34 (1.85, 10.82)^e^	−0.10 (−4.64, 4.45)	0.176
Sensitivity analyses								
Excluding first intervention year data^f^	−5.11 (−9.50, −0.70)^d^	1.31 (−3.13, 5.77)	−4.30 (−8.61, 0.01)	−2.21 (−6.64, 2.23)	−2.90 (−9.12, 3.32)	3.52 (−2.90, 9.95)	−2.09 (−8.38, 4.20)	0.542
Per protocol^g^	−5.95 (−9.17, −2.73)^c^	2.54 (−0.53, 5.62)	−3.89 (−7.05, −0.72)^d^	−3.84 (−6.96, −0.73)^d^	−2.11 (−6.63, 2.41)	6.39 (1.91, 10.86)^e^	−0.04 (−4.57, 4.48)	0.178

^a^Unadjusted linear mixed models included price increase, price label, interaction price increase x price label (22 July 2019 to 8 March 2020)

^b^Adjusted linear mixed model further including mean outcome during baseline period (22 July 2019 to 8 March 2020), number of days semester in session, number of days COVID-19 stay-at-home orders in place, location within student residential area, and season

^c^*p* < 0.001

^d^*p* < 0.05

^e^*p* < 0.01

^f^Adjusted linear mixed model excluding sales data from 9 March 2020 to 7 March 2021

^g^Adjusted linear mixed model using actual label and pricing conditions received for two machines that incorrectly did not receive any intervention during the trial period

Sensitivity analysis for the primary outcome are included in Table 2. No significant condition effects on the percentage of ‘red’ drink volume sold were identified when excluding sales from the first year of trial (9 March 2020 to 7 March 2021) when COVID-19 stay-at-home orders were in place, although a non-significant difference between ‘Price Label Only’ vs ‘Control’ consistent with the main analysis remained. Using a per protocol analysis (excluding two machines that did not receive the allocated intervention) did not change the main analysis conclusions with the only significant comparison being ‘Price Label Only’ vs Control condition (6.39 percentage point increase, 95%CI 1.91, 10.86, *p* = 0.005).

### Secondary outcomes

Table S1 shows the adjusted comparison between intervention conditions and control condition for secondary outcomes. The ‘Price Label Only’ condition was associated with a 5.86 percentage point (95% CI 1.42, 10.29) increase in the change from baseline for total drink units sold classified as ‘red’; a 0.88g/100mL (0.36, 1.40) increase from baseline total sugar content of drinks sold; and a 16.4kJ/100mL (6.66, 26.1) increase from baseline in energy sold from drinks (all relative to Control condition). Interaction effects between price × label were non-significant for secondary outcomes (*p* > 0.05), except for the sugar content of drinks (interaction effect = −0.78 g/100mL [−1.48, −0.07]) and the energy content of drinks (interaction effect = −16.49 kJ/100mL [−29.81, −3.17]). These negative interactions suggest that the effect of price labelling and price increases may counteract each other, or that the effect of price labels decreases in the presence of a price increase.

No other differences in secondary outcomes between treatment groups reached statistical significance, including change from baseline in: total volume of drinks sold; percentage of ‘amber’ and ‘green’ drinks sold by volume; total drink units sold; percentage of ‘amber’ and ‘green’ drink units; machine revenue from drinks (AUD); machine revenue from snack and drinks (AUD); or number of ‘red’, ‘amber’ and ‘green’ snack units sold (Table S1).

## Discussion

This randomised controlled trial found no independent effect of a 20% price increase on unhealthy drinks and no combined effect of that price increase and price labelling on the healthiness of customer purchases or revenue in university vending machines. Contrary to our hypothesis of no price label effect when used alone, prominent price labelling alone was associated with a 6.34 percentage point increase in percentage of ‘red’ drinks sold by volume per machine per week, compared to the Control group. Differences between the ‘Price Label Only’ group and the Control group also included: decreases in the proportion of ‘green’ drinks sold by volume and by number of units; and increases in the proportions of total drink units sold classified as ‘red’, and energy and total sugar content of drinks sold. No other secondary outcomes reached statistical significance. The effect of ‘Price Label Only’ on ‘red’ drink volume sold remained in a sensitivity analysis using actual machine conditions, and was in the same direction (though non-significant) when excluding the first year of the trial, a period when there were very low machine sales.

Our finding of no impact of a less healthy drink price increase on purchasing unhealthy drinks or healthy substitutes is contrary to our hypothesis and adds further complexity to findings from the broader vending pricing literature. While no peer-reviewed research has tested the impact of a less healthy drink price increase in a university vending machine setting, evidence from SSB tax implementation has shown that purchasing tends to decrease proportionally to price increase (e.g., a 10% price increase is associated with approximately 10% reduction in purchases) [10]. Our findings run contrary to this general SSB tax trend. From the vending intervention literature, a non-randomised trial of a multicomponent intervention with a 25% price increase on chocolate bars combined with changes to product display, availability and point-of-purchase nutrition labelling found an increased percentage of healthier options purchased with no revenue changes [7]. Bos et al. [26] found in a real-world task to select a snack and a drink in university vending, that a 33% price increase on high-calorie products was associated with an increase in low-calorie snack selections but not low-calorie drink selections, compared to a control group. While study methodologies differed, the smaller 20% price changes in the current trial compared to the 25–33% increase in previous studies may have been insufficient to prompt changes in consumer purchasing and is a useful finding to inform future vending initiatives. Although we did not routinely collect data on prices of all products during the trial, most ‘green’ drinks available were more expensive than ‘amber’ and ‘red’ drinks with or without the pricing intervention (Fig. 1).

Highlighting the fact that less healthy drinks were actually cheaper than healthier drinks regardless of whether a price increase had been applied may be a contributor to our unexpected finding that prominent price labelling was associated with an increase in purchases of less healthy drinks. In evaluating product prices, customers usually compare current prices to either previous experiences of that product, or to prices of other items presented at the same time [14]. When the gap between the current price and the “reference price” reaches a certain threshold, this prompts action- i.e. a change in purchasing behaviour [27]. Price labelling may have made comparison of prices between products more explicit, in a setting where prices are often not displayed until the product is selected for purchase (which was the case for all machines in our ‘Control’ and ‘Price Increase Only’ conditions). With price labelling highlighting that less healthy options were actually cheaper than healthy items, this may have promoted purchasing in the opposite intended direction (from a public health perspective). This explanation is supported by the negative interaction between price label and price increase for some outcomes (sugar content of drinks, and energy content of drinks) suggesting the impact of price labelling was mitigated by price increases (i.e. price increase and labelling effects worked in opposite directions). The price increase used in the study may therefore have needed to be considerably greater to see a statistically significant change in purchasing, especially given that the presence of price labels was likely to be necessary for any price increase to be effective. Lower consumer price sensitivity, driven by expectations of higher prices and the convenience nature of vending machine use [14] also suggest that future research should examine the impact of larger price increases in these settings. For public health practice, our findings emphasise the importance of ensuring that healthy alternatives are cheaper than less healthy options, and that this information is clearly displayed to customers at the point-of-selection.

Aligned with previous vending research attempting to isolate the impact of price changes over other point-of-sale interventions [6], we did not find that price increases alone were associated with a change in vending machine purchasing. We determined there was also no effect with price increase and price labelling were used together. We were unable to determine the effect of simultaneously changing pricing and product availability as the machines in our study had been compliant with a multicomponent healthy vending policy for 17 months prior the pricing trial, including altering display to no more than 20% ‘red’ drinks and at least 50% ‘green’ drinks; machine traffic light labelling; and health-promoting machine branding. In our study, those most sensitive to health-motivated pricing changes may have already responded to the previous health-motivated changes to vending already applied by the university (including availability changes), reaching saturation for potential changes to consumer purchasing behaviour. An uncontrolled natural experiment focused on improving the availability of healthy drinks at a different university in Victoria, Australia (by the same lead author) found that implementation of a multicomponent vending policy (to achieve voluntary state government traffic light recommendations) was associated with no change in vending sales of ‘red’ drinks, but with large increases in the sales of ‘green’ (121% increase) and ‘amber’ drinks sold (223%) and an 89% increase in revenue [28]. Although multi-component strategies have been recommended in the literature, our findings suggest the components included and how they are specified need to be very carefully considered [29]. Future research should investigate whether different aspects of multicomponent vending interventions differentially impact purchasing behaviour of different SSB consumer groups.

The absence of revenue changes across all intervention conditions in our study makes sense given the minimal shift in products purchased. The favourable changes in healthy purchases seen in two previous university vending pricing interventions were associated with increased revenue [6], and no changes in revenue and increased profit [7], respectively. The limited evidence to date suggests that health-driven pricing interventions in vending may not be harmful for business profitability.

### Strengths and limitations

Our study used a cluster-randomised controlled trial design. Our six-monthly audits detected that the allocated treatment condition was implemented accurately in all but two machines with ‘per protocol’ analysis matching the analysis where these machines were excluded.

We know that the dramatic changes in sales related to COVID-19 coinciding with the trial start were due to huge changes in the actual numbers and characteristics of students and staff on campus. Because detailed attendance figures were not available, our analysis used semester dates and ‘stay-at-home’ orders as proxy covariates to adjust for changes in the size of the population of potential vending customers. Future studies could explore the use of geolocation methods to determine actual student and staff on-campus attendance [30].

At the time the trial was planned, no previous study had collected the data needed to conduct an a priori power calculation. Even if such a calculation had shown that the study was adequately powered to detect meaningful changes in proportion of ‘red’ drink sold (main outcome), it would have been based on pre-COVID-19 estimates. The dramatic shifts in sales patterns caused by the COVID-19 restrictions would likely have made those calculations invalid for the level of sales observed during our intervention period.

Vending machines were not randomised individually but rather grouped into clusters to prevent “contamination” between machines located in close proximity and assigned to different conditions. Although we attempted to balance multiple factors when creating the clusters, unaccounted-for or unconsidered factors related to the consumer population and their behaviours may have confounded the observed effects of the intervention conditions.

Finally, we assumed that nothing was sold in a vending machine in a given week if data were missing for one or more weeks between the start and end dates that the machine was known to be operating. As sales data is generated automatically and no data is recorded when no sale is made, it was not possible to verify whether data were missing or nothing was sold for those weeks.

### Generalisability

In line with the dramatic falls in overall sales in the university vending machines in our trial coinciding with the start of the COVID-19 pandemic, the retail value of Australian alcoholic drinks, non-alcoholic drinks and tobacco vending declined approximately 61% from 2019–20 (food vending declined 5%), and fell a further 8% during 2021 (food vending declined 5%) [31]. Australian sales for both categories began increasing again from 2022. Repetition of the study in post-pandemic conditions and with different inflationary pressures may produce different consumer responses to the intervention.

Differences in population SSB consumption patterns; vending machine price labelling practices, offerings and usage patterns; local nutrition policies; COVID-19 stay-at-home orders; and price responsiveness may affect study generalisability to other countries. The mean Australian SSB intake (3.1 (95%UI 2.5–3.8) 8oz serves/week) is slightly higher than the average global intake (2.7 (95%UI 2.5–2.9) serves/week), however there is high variation between countries [32]. A recent cross-sectional study found substantial differences in SSB price responsiveness between countries, with lower income countries being the most price responsive [33].

It is unclear what the general prevalence of price labelling is on vending machines in Australia or globally, or how the absence of price labelling on machines at baseline may have removed potential ‘anchoring’ effects of prior price salience [15]. Finally, university populations are younger, more highly educated and lower income compared to many other settings [34]. Previous research suggests the younger (university-aged) consumers and older adults (compared to middle age adults) may be most responsive to SSB pricing changes [33].

## Conclusions

This study found no impact on the healthiness of vending machine sales of a 20% price increase on unhealthy drinks, with or without prominent price labelling. It found an increase in sales of less healthy drinks in response to prominent price labelling displayed without price intervention. The unexpected findings may be due to the price increase being too small to have an effect, with less healthy drink options cheaper than healthy drink options even after the increase had been applied. This is the first study to examine the effects of a long-term price increase on unhealthy drinks in vending machines. Future studies should test the impact of relative prices of healthy compared to less healthy options, with and without price labelling, and among different consumer groups in order to determine an effective suite of changes to best balance consumer health and preferences, and vendor financial viability.

## Supplementary Information

Supplementary Material 1. Appendix 1: Audit survey. Supplementary Figure S1: Total drink volume sales over time within each treatment condition (n=60 vending machines) (22 July 2019 to 6 March 2022). Supplementary Table S1: Secondary sales outcomes compared to baseline intervention conditions relative to control condition (n=59 machines)^a^.

## Data Availability

The datasets used during the current study are not available due to a commercial-in-confidence agreement with the vending supplier.

## Abbreviations

ANZCTR: Australian New Zealand Clinical Trials Registry
AUD: Australian Dollar
CI: Confidence Interval
COVID-19: Coronavirus Disease of 2019
RCT: Randomised Controlled Trial
SD: Standard Deviation
SSB: Sugar-Sweetened Beverage
US: United States
