# Supplementary material for: The independent and combined effect of price increase and price labelling on sugar-sweetened beverage vending machine sales in an Australian university: a factorial randomised controlled trial
Source: Int J Behav Nutr Phys Act. 2026 Mar 18;23:44. doi: 10.1186/s12966-026-01890-4 (PMC13126900; doi:10.1186/s12966-026-01890-4)
Supplement: Supplementary file 1 — Supplementary Material 1. Appendix 1: Audit survey. Supplementary Figure S1: Total drink volume sales over time within each treatment condition (n=60 vending machines) (22 July 2019 to 6 March 2022). Supplementary Table S1: Secondary sales outcomes compared to baseline intervention conditions relative to control condition (n=59 machines)a. [file 12966_2026_1890_MOESM1_ESM.docx]

**Supplementary file**

**Appendix 1: Audit survey**

Q1 What is the machine number? Note if there are any discrepancies between number on machine and location- record both and describe location if different from map.

Q2 Is the machine located in a residential facility?

o Yes

o No

Q3 Is the machine accessible to general student population? (if you need a swipe card to access the area select NO)

o Yes

o No

Q4 Are prices labels displayed directly under each item?

o Yes, all items have prices

o Some, but not all items have prices

o No items have prices

Q5 Price of Coke full sugar

o $2.70

o $2.20

o Other (please specify) __________________________________________________

Q6 Price of Coke No sugar

o $2.70

o $2.20

o Other (please specify) __________________________________________________

Q7 Are traffic light labels displayed directly under each item?

o Yes, all items have traffic light labels

o Some, but not all items have traffic light labels

o No items have traffic light labels

Q8 Does the machine have a touch screen?

o Yes

o No

Q9 Is a retail drinks fridge in view from the [removed for blinding] machine? (incl. cafés or restaurants down the hall, upstairs, right next door)

o Yes

o No

Q10 Is there an [removed for blinding] (Asian-style vending machine) within approx. 10m?

o Yes

o No

Q11 Are there any accessible water fountains, sinks or free drinks (e.g. coffee and tea) in view?

o Yes

o No

Q12 Any other comments?

________________________________________________________________

Q13 Take a photo of the [removed for blinding] machine

1. Take photo of the machine number (so will know which photos correspond to which machine)

2. Take photos of machines so that each product (and price is visible). This may mean taking a “large” photo of the whole machine, and then individual photos per row.

3. Check photos after each machine to check that each item and price (if relevant) is visible

Q14 Take a photo of any [removed for blinding] Asian-style vending machines within 10m.

1. Take photo of the machine number (so will know which photos correspond to which machine)

2. Take photos of machines so that each product (and price is visible). This may mean taking a “large” photo of the whole machine, and then individual photos per row.

3. Check photos after each machine to check that each item and price (if relevant) is visible.

**Supplementary Figure S1: Total drink volume sales over time within each treatment condition (n=60 vending machines) (22 July 2019 to 6 March 2022)**

**Supplementary Table S1: Secondary sales outcomes compared to baseline intervention conditions relative to control condition (n=59 machines) ^a^**

| **Outcome (weekly sales)** | **Mean difference** | | | | | | **Interaction**  **Price × Label** |
| --- | --- | --- | --- | --- | --- | --- | --- |
|  | **Price Increase Only** | | **Price Label Only** | | **Price Increase + Price Label** | |  |
|  | **Estimated difference ^b^ (95%CI)** | **p** | **Estimated difference ^b^ (95%CI)** | **p** | **Estimated difference ^b^ (95% CI)** | **p** | **p** |
| **Total drink volume (mL)^c^** | 1339.63 (-666.73, 3346) | 0.191 | 297.38 (-1762.5, 2357.27) | 0.777 | -424.67 (-2457.74, 1608.4) | 0.682 | 0.148 |
| **‘Amber’ drink volume (% total drink volume)** | -2.32 (-8.28, 3.64) | 0.445 | -0.57 (-6.67, 5.52) | 0.854 | 1.48 (-4.68, 7.64) | 0.637 | 0.305 |
| **‘Green’ drink volume (% total drink volume)** | 3.40 (-4.51, 11.32) | 0.399 | -5.69 (-13.93, 2.55) | 0.176 | -1.90 (-10.23, 6.42) | 0.654 | 0.945 |
| **Total drink units** | 311.05 (-203.49, 825.58) | 0.236 | 86.77 (-440.51, 614.05) | 0.747 | -110.34 (-631.15, 410.47) | 0.678 | 0.164 |
| **‘Red’ drink units (% total drink units)** | -2.23 (-6.60, 2.13) | 0.316 | 5.86 (1.42, 10.29) | 0.010 | -0.73 (-5.21, 3.76) | 0.750 | 0.167 |
| **‘Amber’ drink units (% total drink units)** | -2.57 (-8.51, 3.37) | 0.397 | -1.37 (-7.47, 4.72) | 0.658 | 1.01 (-5.13, 7.16) | 0.747 | 0.245 |
| **‘Green’ drink units (% total drink units)** | 3.66 (-3.80, 11.12) | 0.336 | -4.80 (-12.59, 2.98) | 0.227 | -0.99 (-8.85, 6.87) | 0.805 | 0.977 |
| **Energy (kJ/100mL) from drinks ^d^** | 9.21 (-0.35, 18.78) | 0.059 | 16.4 (6.66, 26.15) | 0.001 | 9.13 (-0.45, 18.71) | 0.062 | 0.015 |
| **Total sugar (g/100mL) from drinks ^e^** | 0.15 (-0.35, 0.65) | 0.558 | 0.88 (0.36, 1.40) | 0.001 | 0.25 (-0.26, 0.76) | 0.329 | 0.030 |
| **Total snack units** | 329.42 (-83.08, 741.93) | 0.118 | 93.79 (-323.42, 510.99) | 0.660 | -41.10 (-455.78, 373.58) | 0.846 | 0.108 |
| **‘Red’ snack units (% total snack units)** | -1.29 (-6.60, 4.03) | 0.635 | 4.78 (-0.55, 10.12) | 0.079 | -0.49 (-5.89, 4.91) | 0.859 | 0.290 |
| **‘Amber’ snack units (% total snack units)** | -1.79 (-5.99, 2.41) | 0.404 | -4.36 (-8.69, -0.03) | 0.049 | -2.93 (-7.29, 1.44) | 0.189 | 0.285 |
| **‘Green’ snack units sold (% total snack units)** | 1.57 (-1.38, 4.53) | 0.297 | -1.08 (-4.03, 1.87) | 0.472 | 2.26 (-0.71, 5.23) | 0.136 | 0.407 |
| **Machine revenue from drinks (AUD)** | 10.57 (-4.17, 25.30) | 0.160 | 1.74 (-13.38, 16.86) | 0.822 | -2.56 (-17.49, 12.37) | 0.737 | 0.155 |
| **Machine revenue from snack and drinks (AUD)** | 19.25 (-5.93, 44.44) | 0.134 | 4.55 (-21.31, 30.40) | 0.730 | -4.06 (-29.62, 21.50) | 0.755 | 0.118 |

^a^ Linear mixed models comparisons between intervention conditions and control. Adjusted models for mean outcome during baseline period (22 July 2019 to 8 March 2020), number of days semester in session, number of days COVID-19 stay-at-home orders in place, location within student residential area, and season. ^b^ Estimated difference compared to Control condition. ^c^ To convert to fluid ounces, divide by 29.57; ^d^ To convert to kilocalories, divide by 4.184; ^e^ To convert to ounces, divide by 28.35.
